# Aflatoxins: A Global Concern for Food Safety, Human Health and Their Management

**DOI:** 10.3389/fmicb.2016.02170

**Published:** 2017-01-17

**Authors:** Pradeep Kumar, Dipendra K. Mahato, Madhu Kamle, Tapan K. Mohanta, Sang G. Kang

**Affiliations:** ^1^Department of Forestry, North Eastern Regional Institute of Science and TechnologyNirjuli, India; ^2^Division of Food Science & Postharvest Technology, Indian Agricultural Research InstituteNew Delhi, India; ^3^Department of Biotechnology, Yeungnam UniversityGyeongsan, South Korea

**Keywords:** aflatoxins, health issues, *Aspergillus* sp., secondary metabolites, food contamination

## Abstract

The aflatoxin producing fungi, *Aspergillus spp.*, are widely spread in nature and have severely contaminated food supplies of humans and animals, resulting in health hazards and even death. Therefore, there is great demand for aflatoxins research to develop suitable methods for their quantification, precise detection and control to ensure the safety of consumers’ health. Here, the chemistry and biosynthesis process of the mycotoxins is discussed in brief along with their occurrence, and the health hazards to humans and livestock. This review focuses on resources, production, detection and control measures of aflatoxins to ensure food and feed safety. The review is informative for health-conscious consumers and research experts in the fields. Furthermore, providing knowledge on aflatoxins toxicity will help in ensure food safety and meet the future demands of the increasing population by decreasing the incidence of outbreaks due to aflatoxins.

## Introduction

**FIGURE 1:**
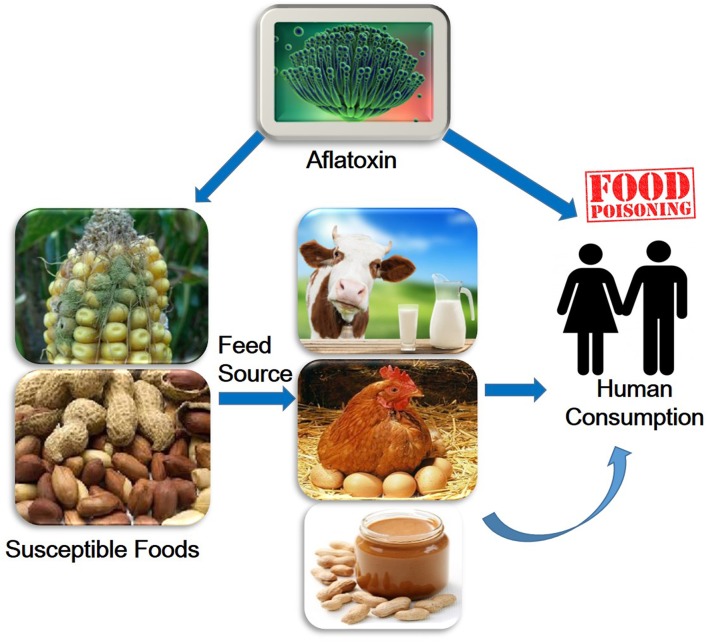
**Overview of aflatoxin effects on humans**.

Aflatoxins are one of the highly toxic secondary metabolites derived from polyketides produced by fungal species such as *Aspergillus flavus, A. parasiticus*, and *A. nomius* (Payne and Brown, 1998). These fungi usually infect cereal crops including wheat, walnut, corn, cotton, peanuts and tree nuts (Jelinek et al., 1989; Severns et al., 2003), and can lead to serious threats to human and animal health by causing various complications such as hepatotoxicity, teratogenicity, and immunotoxicity (**Figure 1**) (Amaike and Keller, 2011; Kensler et al., 2011; Roze et al., 2013). The major aflatoxins are B1, B2, G1, and G2, which can poison the body through respiratory, mucous or cutaneous routes, resulting in overactivation of the inflammatory response (Romani, 2004).

Food safety is one of the major problems currently facing the world; accordingly, a variety of studies have been conducted to discuss methods of addressing consumer concerns with various aspects of food safety (Nielsen et al., 2009). Since 1985, the United States Food and Drug Administration (USFDA) has restricted the amount of mycotoxins permitted in food products. The USDA Grain and Plant Inspection Service (GPIS) have implemented a service laboratory for inspection of mycotoxins in grains. Additionally, the Food and Agricultural Organization (FAO) and World Health Organization (WHO) have recognized many toxins present in agricultural products. When mycotoxins are contaminated into foods, they cannot be destroyed by normal cooking processes. However, there have been many recent advances in food processing developed to keep final food products safe and healthy, such as hazard analysis of critical control points (HACCP) and good manufacturing practices (GMP; Lockis et al., 2011; Cusato et al., 2013; Maldonado-Siman et al., 2014). Moreover, several physical, chemical and biological methods can be applied to partially or completely eliminate these toxins from food and guarantee the food safety and health concerns of consumers. This review provides an overview of aflatoxigenic fungi, chemistry and biosynthesis of aflatoxins, along with their diversity in occurrence, and their health related risks to humans and livestock. Moreover, the effects of processing techniques on aflatoxins and various physical, chemical and biological methods for their control and management in food are discussed briefly.

## Outbreaks Due to Aflatoxins

In 1974, a major outbreak of hepatitis due to aflatoxin was reported in the states of Gujrat and Rajasthan in India, resulting in an estimated 106 deaths (Krishnamachari et al., 1975). The outbreak lasted for 2 months and was confined to tribal people whose main staple food, maize, was later confirmed to contain aflatoxin. The preliminary analysis confirmed that consumption of *A. flavus* had occurred (Krishnamachari et al., 1975; Bhatt and Krishnamachari, 1978). Another outbreak of aflatoxin affecting both humans and dogs was reported in northwest India in 1974 (Tandon et al., 1977; Bhatt and Krishnamachari, 1978; Reddy and Raghavender, 2007). A major aflatoxin exposure outbreak was subsequently documented in Kenya in 1981 (Ngindu et al., 1982). Since 2004, multiple aflatoxicosis outbreaks have been reported worldwide, resulting in 500 acute illness and 200 deaths (Centers for Disease Control and Prevention [CDCP], 2004; Azziz-Baumgartner et al., 2005). Most outbreaks have been reported from rural areas of the East Province of Kenya in 2004 and occurred because of consumption of home grown maize contaminated with molds. Preliminary testing of food from affected areas revealed the presence of aflatoxin as reported in 1981 (Ngindu et al., 1982). In 2013, countries in Europe including Romania, Serbia, and Croatia reported the nationwide contamination of milk with aflatoxin^1^.

## Major Source of Aflatoxin

The major sources of aflatoxins are fungi such as *A. flavus, A. parasiticus*, and *A. nomius* (Kurtzman et al., 1987), although they are also produced by other species of *Aspergillus* as well as by *Emericella* spp. (Reiter et al., 2009). There are more than 20 known aflatoxins, but the four main ones are aflatoxin B1 (AFB1), aflatoxin B2 (AFB2), aflatoxin G1 (AFG1), and aflatoxin G2 (AFG2; Inan et al., 2007), while aflatoxin M1 (AFM1) and M2 (AFM2) are the hydroxylated metabolites of AFB1 and AFB2 (Giray et al., 2007; Hussain and Anwar, 2008).

## *Aspergillus* spp.

The *Aspergillus* species are an industrially important group of microorganisms distributed worldwide. *A. niger* has been given Generally Recognized as Safe (GRAS) status by the USFDA (Schuster et al., 2002). However, some species have negative impacts and cause diseases in grape, onion, garlic, peanut, maize, coffee, and other fruits and vegetables (Lorbeer et al., 2000; Magnoli et al., 2006; Waller et al., 2007; Rooney-Latham et al., 2008). Moreover, *Aspergillus* section *nigri* produces mycotoxins such as ochratoxins and fumonisins in peanut, maize, and grape (Astoreca et al., 2007a,b; Frisvad et al., 2007; Mogensen et al., 2009).

Plant–pathogen interactions have been studied using molecular markers such as green fluorescent protein (GFP) isolated from *Aequorea victoria* (Prasher et al., 1992). The GFP gene has been successfully inserted into *Undifilum oxytropis* (Mukherjee et al., 2010), *Fusarium equiseti* (Macia-Vicente et al., 2009), and *Muscodor albus* (Ezra et al., 2010) and utilized to study the expression of different proteins and production of mycotoxins. *A*. *flavus* and *A*. *parasiticus* infect many crops in the field, during harvest, in storage, and during processing. *A*. *flavus* is dominant in corn, cottonseed, and tree nuts, whereas *A*. *parasiticus* is dominant in peanuts. *A. flavus* consists of mycelium, conidia, or sclerotia and can grow at temperatures ranging between 12 and 48°C (Hedayati et al., 2007). *A*. *flavus* produces AFBI and AFB2, whereas *A*. *parasiticus* isolates produce AFGI, AFG2, AFM1, AFBI, and AFB2. *A*. *flavus* produces a number of airborne conidia and propagules that infect plants such as cotton (Lee et al., 1986). A high number of propagules was reported in soil, air, and on cotton leaves during mid- to late August, while soilborne inoculum increased drastically between April and December in cotton fields in Arizona (Ashworth et al., 1969). This fungus can even colonize moribund rye cover crop and peanut fruit debris (Griffin and Garren, 1976).

## Aflatoxin (AFT)

Among the mycotoxins affecting food and feed, aflatoxin is the major one in food that ultimately harms human and animal health (Boutrif, 1998). The level of toxicity associated with aflatoxin varies with the types present, with the order of toxicity being AFTs-B_1_ > AFTs-G_1_ > AFTs-B_2_ > AFTs-G_2_ (Jaimez et al., 2000).

## Chemistry and Biosynthesis of Aflatoxins

Chemically, aflatoxins (AFTs) are difuranocoumarin derivatives in which a bifuran group is attached at one side of the coumarin nucleus, while a pentanone ring is attached to the other side in the case of the AFTs and AFTs-B series, or a six-membered lactone ring is attached in the AFTs-G series (Bennett and Klich, 2003; Nakai et al., 2008). The physical, biological and chemical conditions of *Aspergillus* influence the production of toxins. Among the 20 identified AFTs, AFT-B_1_, and AFT-B_2_ are produced by *A. flavus*, while AFT-G_1_ and AFT-G_2_ along with AFT-B_1_ and AFT-B_2_ are produced by *A. parasiticus* (Bennett and Klich, 2003). AFT-B_1_, AFT-B_2_, AFT-G_1_, and AFT-G_2_ are the four major naturally produced aflatoxins (Pitt, 2000). AFTs-M_1_ and AFTs-M_2_ are derived from aflatoxin B types through different metabolic processes and expressed in animals and animal products (Weidenborner, 2001; Wolf-Hall, 2010). AFT-B_1_ is highly carcinogenic (Squire, 1981), as well as heat resistant over a wide range of temperatures, including those reached during commercial processing conditions (Sirot et al., 2013).

The biosynthetic pathway of aflatoxins consists of 18 enzymatic steps for conversion from acetyl-CoA, and at least 25 genes encoding the enzymes and regulatory pathways have been cloned and characterized (Yu et al., 2002; Yabe and Nakajima, 2004). The gene comprises 70 kb of the fungal genome and is regulated by the regulatory gene, *aflR* (Yabe and Nakajima, 2004; Yu et al., 2004; Price et al., 2006). The metabolic grid involved in the aflatoxin biosynthesis (Yabe et al., 1991, 2003). Hydroxyversicolorone (HVN) is converted to versiconal hemiacetal acetate (VHA) by a cytosol monooxygenase, in which NADPH is a cofactor (Yabe et al., 2003). Monooxygenase is encoded by the *moxY* gene, which catalyzes the conversion of HVN to VHA and the accumulation of HVN and versicolorone (VONE) occurs in the absence of the *moxY* gene (Wen et al., 2005).

## Gene Responsible for Aflatoxin Production

Various genes and their enzymes are involved in the production of sterigmatocystin (ST) dihydrosterigmatocystin (DHST), which are the penultimate precursors of aflatoxins (Cole and Cox, 1987). The aflatoxin biosynthesis gene *nor-1*, which was first cloned in *A. Parasiticus*, is named after the product formed by the gene during biosynthesis (Chang et al., 1992). These genes named according to substrate and the product formed *nor-1* (norsolorinic acid [NOR]), *norA*, *norB, avnA* (averanti [AVN]), *avfA* (averufin [AVF]), *ver-1* (versicolorin A [VERA]), *verA* and *verB* while those based on enzyme functions *fas-2* (FAS alpha subunit), *fas-1* (FAS beta subunit), *pksA* (PKS), *adhA* (alcohol dehydrogenase), *estA* (esterase), *vbs* (VERB synthase), *dmtA* (mt-I; *O*-methyltransferase I), *omtA* (*O*-methyltransferase A), *ordA* (oxidoreductase A), *cypA* (cytochrome P450 monooxygenase), *cypX* (cytochrome P450 monooxygenase), and *moxY* (monooxygenase). Initially, the aflatoxin regulatory gene was named *afl-2* in *A. flavus* (Payne et al., 1993) and *apa-2* in *A. parasiticus* (Chang et al., 1993). However, it was subsequently referred to as *aflR* in *A. flavus, A. parasiticus*, and *A. nidulans* because of its role as a transcriptional activator. Previous studies have shown that *aflA* (fas-2), *aflB* (*fas-1*), and *aflC* (*pksA*) are responsible for the conversion of acetate to NOR (Townsend et al., 1984; Brown et al., 1996). Moreover, the *uvm8* gene was shown to be essential for NOR biosynthesis as well as aflatoxin production in *A. parasiticus.* The amino acid of sequence of the gene is similar to that of the beta subunit of FASs *(FAS1)* from *Saccharomyces cerevisiae* (Trail et al., 1995a,b). FAS forms the polyketide backbone during aflatoxin synthesis; hence, the *uvm8* gene was named *fas-1* (Mahanti et al., 1996). Fatty acid syntheses (FASs) is responsible for sterigmatocystin (ST) biosynthesis in *A. nidulans* and further identified two genes viz., *stcJ* and *stcK* that encode FAS and FAS subunits (FAS-2 and FAS-1; Brown et al., 1996).

## Occurrence in Food

Aflatoxins are found in various cereals, oilseeds, spices, and nuts (Lancaster et al., 1961; Weidenborner, 2001; Reddy, 2010; Iqbal et al., 2014). These *Aspergillus* colonize among themselves and produce aflatoxins, which contaminate grains and cereals at various steps during harvesting or storage. Fungal contamination can occur in the field, or during harvest, transport and storage (Kader and Hussein, 2009). Aflatoxins contamination of wheat or barley is commonly happen by the result of inappropriate storage (Jacobsen, 2008). In milk, aflatoxins is generally at 1–6% of the total content in the feedstuff (Jacobsen, 2008). AFTs infect humans following consumption of aflatoxins contaminated foods such as eggs, meat and meat products, milk and milk products, (Bennett and Klich, 2003; Piemarini et al., 2007).

## Effects on Agriculture and Food

Mycotoxins, including aflatoxin, have affected most crops grown worldwide; however, the extent of aflatoxin toxicity varies according to the commodities (Abbas et al., 2010). Aflatoxin can infect crops during growth phases or even after harvesting (Kumar et al., 2008). Exposure to this toxin poses serious hazards to human health (Umoh et al., 2011). Commodities such as corn, peanuts, pistachio, Brazil nuts, copra, and coconut are highly prone to contamination by aflatoxin (Idris et al., 2010; Cornea et al., 2011), whereas wheat, oats, millet, barley, rice, cassava, soybeans, beans, pulses, and sorghum are usually resistant to aflatoxin contamination. However, agricultural products such as cocoa beans, linseeds, melon seeds and sunflower seeds are seldom contaminated (Bankole et al., 2010). Aflatoxin was on the Rapid Alert System for Food and Feed (RASFF) of the European Union in 2008 because of its severe effects (European Commission, 2009), and the International Agency for Research on Cancer (IARC) later categorized AFB1 as a group I carcinogen for humans (Seo et al., 2011). Despite several research and control measures, aflatoxin is still a major threat to food and agricultural commodities.

## Mechanism of Toxicity and Health Effects by Aflatoxin

Aflatoxin are specifically target the liver organ (Abdel-Wahhab et al., 2007). Early symptoms of hepatotoxicity of liver caused by aflatoxins comprise fever, malaise and anorexia followed with abdominal pain, vomiting, and hepatitis; however, cases of acute poisoning are exceptional and rare (Etzel, 2002). Chronic toxicity by aflatoxins comprises immunosuppressive and carcinogenic effects. Evaluation of the effects of AFT-B_1_ on splenic lymphocyte phenotypes and inflammatory cytokine expression in male F344 rats have been studied (Qian et al., 2014). AFT-B_1_ reduced anti-inflammatory cytokine IL-4 expression, but increased the pro-inflammatory cytokine IFN-γ and TNF-α expression by NK cells. These findings indicate that frequent AFT-B_1_ exposure accelerates inflammatory responses via regulation of cytokine gene expression. Furthermore, Mehrzad et al. (2014) observed that AFT-B_1_ interrupts the process of antigen-presenting capacity of porcine dendritic cells, suggested this perhaps one of mechanism of immunotoxicity by AFT-B_1_.

Aflatoxins cause reduced efficiency of immunization in children that lead to enhanced risk of infections (Hendrickse, 1997). The hepatocarcinogenicity of aflatoxins is mainly due to the lipid peroxidation and oxidative damage to DNA (Verma, 2004). AFTs-B_1_ in the liver is activated by cytochrome p450 enzymes, which are converted to AFTs-B1-8, 9-epoxide, which is responsible for carcinogenic effects in the kidney (Massey et al., 1995). Among all major mycotoxins, aflatoxins create a high risk in dairy because of the presence of their derivative, AFTs-M_1_, in milk, posing a potential health hazard for human consumption (Van Egmond, 1991; Wood, 1991). AFTs-B_1_ is rapidly absorbed in the digestive tract and metabolized by the liver, which converts it to AFT-M_1_ for subsequent secretion in milk and urine (Veldman et al., 1992). Although AFTs-M_1_ is less mutagenic and carcinogenic than AFTs-B_1_, it exhibits high genotoxic activity. The other effects of AFTs-M_1_ include liver damage, decreased milk production, immunity suppression and reduced oxygen supply to tissues due to anemia (Aydin et al., 2008), which reduces appetite and growth in dairy cattle (Akande et al., 2006). Several studies have shown the detrimental effects of aflatoxins exposure on the liver (Sharmila Banu et al., 2009), epididymis (Agnes and Akbarsha, 2001), testis (Faisal et al., 2008), kidney and heart (Mohammed and Metwally, 2009; Gupta and Sharma, 2011). It has been found that aflatoxin presences in post-mortem brain tissue (Oyelami et al., 1995), suggested that its ability to cross the blood brain barrier (Qureshi et al., 2015). AFTs also cause abnormalities in the structure and functioning of mitochondrial DNA and brain cells (Verma, 2004). The effects of aflatoxin on brain chemistry have been reviewed in details by Bbosa et al. (2013). Furthermore, few reports have described the effects of AFTs-B_1_ administration on the structure of the rodent central nervous system (Laag and Abdel Aziz, 2013).

The liver toxicology of aflatoxin is also a critical issue (IARC, 2002; Iqbal et al., 2014). Limited doses are not harmful to humans or animals; however, the doses that do cause-effects diverse among Aflatoxin groups. The expression of aflatoxin toxicity is regulated by factors such as age, sex, species, and status of nutrition of infected animals (Williams et al., 2004). The symptoms of acute aflatoxicosis include oedema, haemorrhagic necrosis of the liver and profound lethargy, while the chronic effects are immune suppression, growth retardation, and cancer (Gong et al., 2004; Williams et al., 2004; Cotty and Jaime-Garcia, 2007).

## Effects of Processing on Aflatoxin

Techniques to eliminate aflatoxin may be either physical or chemical methods. Removing mold-damaged kernels, seeds or nuts physically from commodities has been observed to reduce aflatoxins by 40–80% (Park, 2002). The fate of aflatoxin varies with type of heat treatment (e.g., cooking, drying, pasteurization, sterilization, and spray drying; Galvano et al., 1996). Aflatoxins decompose at temperatures of 237–306°C (Rustom, 1997); therefore, pasteurization of milk cannot protect against AFM1 contamination. Awasthi et al. (2012) reported that neither pasteurization nor boiling influenced the level of AFM1 in bovine milk. However, boiling corn grits reduced aflatoxins by 28% and frying after boiling reduced their levels by 34–53% (Stoloff and Trucksess, 1981). Roasting pistachio nuts at 90°C, 120°C, and 150°C for 30, 60 and 120 min was found to reduce aflatoxin levels by 17–63% (Yazdanpanah et al., 2005). The decrease in aflatoxin content depends on the time and temperature combination. Moreover, alkaline cooking and steeping of corn for the production of tortillas reduces aflatoxin by 52% (Torres et al., 2001). Hameed (1993) reported reductions in aflatoxin content of 50–80% after extrusion alone. When hydroxide (0.7 and 1.0%) or bicarbonate (0.4%) was added, the reduction was enhanced to 95%. Similar results were reported by Cheftel (1989) for the extrusion cooking of peanut meal. The highest aflatoxin reduction was found to be 59% with a moisture content of 35% in peanut meal, and the extrusion variables non-significantly affected its nutritional composition (Saalia and Phillips, 2011a). Saalia and Phillips (2011b) reported an 84% reduction in aflatoxin of peanut meal when cooked in the presence of calcium chloride.

## Effects of Environmental Temperature on Aflatoxin Production

Climate change plays a major role in production of aflatoxin from *Aspergillus* in food crops (Paterson and Lima, 2010, 2011; Magan et al., 2011; Wu F. et al., 2011; Wu S. et al., 2011). Climate change affects the interactions between different mycotoxigenic species and the toxins produced by them in foods and feeds (Magan et al., 2010; Paterson and Lima, 2012). Changes in environmental temperature influence the expression levels of regulatory genes (*aflR* and *aflS*) and aflatoxin production in *A. flavus* and *A. parasiticus* (Schmidt-Heydt et al., 2010, 2011). A good correlation between the expression of an early structural gene (*aflD*) and AFB1 has been reported by Abdel-Hadi et al. (2010). Temperature interacts with water activity (a_w_) and influences the ratio of regulatory genes (*aflR*/*aflS*), which is directly proportional to the production of AFB1 (Schmidt-Heydt et al., 2009, 2010). The interactions between water activity and temperature have prominent effect on *Aspergillus* spp. and aflatoxin production (Sanchis and Magan, 2004; Magan and Aldred, 2007). Increasing the temperature to 37°C and water stress significantly reduces the production of AFB1 produced, despite the growth of *A. flavus* under these conditions. The addition of CO_2_ under the same temperature and water activity enhances AFB1 production (Medina et al., 2014). According to Gallo et al. (2016), fungal biomass and AFB1 production were reported to be highest at 28°C and 0.96 *a*_w_, while no fungal growth or AFB1 production was seen at 20°C with *a*_w_ values of 0.90 and 0.93. There was also no AFB1 production observed at 37°C. Reverse transcriptase quantitative PCR also revealed that the regulatory genes *aflR* and *aflS* were highly expressed at 28°C, while the lowest expression was observed at 20 and 37°C, suggesting that temperature plays a significant role in gene expression and aflatoxin production (Gallo et al., 2016).

## Detection Techniques

The detection and quantification of aflatoxin in food and feed is a very important aspect for the safety concerns. Aflatoxins are usually detected and identified according to their absorption and emission spectra, with peak absorbance occurring at 360 nm. B toxins exhibit blue fluorescence at 425 nm, while G toxins show green fluorescence at 540 nm under UV irradiation. This florescence phenomenon is widely accepted for aflatoxins. Thin layer chromatography (TLC) is among one of the oldest techniques used for aflatoxin detection (Fallah et al., 2011), while high performance liquid chromatography (HPLC), liquid chromatography mass spectroscopy (LCMS), and enzyme linked immune-sorbent assay (ELISA) are the methods most frequently used for its detection (Tabari et al., 2011; Andrade et al., 2013; Sulyok et al., 2015). ELISA can be used to identify aflatoxins based on estimation of AfB1-lysine (metabolite of AFB1 toxin) concentration in the blood. Specifically, the test detects levels of AfB1 in blood as low as 5 pg/mg albumin, making it a cost effective method for routine monitoring that can also be utilized for the detection of hepatitis B virus. Room temperature phosphorescence (RTP) in aflatoxigenic strains grown on media is commonly used in food mycology. Aflatoxins immobilized on resin beads can induce RTP in the presence or absence of oxygen and heavy atoms (Costa-Fernandez and Sanz-Medel, 2000) and also have high sensitivity and specificity (Li et al., 2003). Moreover, several biosensors and immunoassays have been developed to detect ultra-traces of aflatoxins to ensure the food safety.

## Degradation Kinetics

Various treatments including chemical, physical, and biological methods are routinely utilized for effective degradation, mitigation and management of aflatoxin (Shcherbakova et al., 2015). The aflatoxins AFB1 and AFG1 are completely removed by ozone treatment at 8.5–40 ppm at different temperatures, but AFB2 and AFG2 are not affected by this method. The degradation of aflatoxin followed first order kinetic equation. However, microbial and enzymatic degradation is preferred for the biodegradation of aflatoxin due to its eco-friendly nature (Agriopoulou et al., 2016). The bacterium *Flavobacterium aurantiacum* reportedly removes AFM1 from milk and *Nocardia asteroides* transforms AFB1 to fluorescent product (Wu et al., 2009). *Rhodococcus* species are able to degrade aflatoxins (Teniola et al., 2005) and their ability to degrade AFB1 occurs in the following order: *R. ruber* < *R. globerulus* < *R. coprophilus* < *R. gordoniae* < *R. pyridinivorans* and < *R. erythropolis* (Cserhati et al., 2013). Fungi such as *Pleurotus ostreatus, Trametes versicolor, Trichosporon mycotoxinivorans, S. cerevisiae, Trichoderma* strains, and *Armillariella tabescens* are known to transform AFB1 into less toxic forms (Guan et al., 2008). Zhao et al. (2011) reported purification of extracellular enzymes from the bacterium *Myxococcus fulvus* ANSM068 with a final specific activity of 569.44 × 103 U/mg. The pure enzyme (100 U/mL) had a degradation ability of 96.96% for AFG1 and 95.80% for AFM1 after 48 h of incubation. Moreover, the recombinant laccase produced by *A. niger* D15-Lcc2#3 (118 U/L) was found to lead to a decrease in AFB1 of 55% within 72 h (Alberts et al., 2009).

## Management and Control Strategies

The biocontrol principle of competitive exclusion of toxigenic strains of *A. flavus* involves the use of non-toxigenic strains to reduce aflatoxin contamination in maize (Abbas et al., 2006). The use of biocontrol agents such as *Bacillus subtilis*, *Lactobacillus* spp., *Pseudomonas* spp., *Ralstonia* spp., and *Burkholderia* spp. are effective at control and management of aflatoxins (Palumbo et al., 2006). Several strains of *B. subtilis* and *P. solanacearum* isolated from the non-rhizosphere of maize soil have been reported to eliminate aflatoxin (Nesci et al., 2005). Biological control of aflatoxin production in crops in the US has been approved by the Environmental Protection Agency and two commercial products based on atoxigenic *A. flavus* strains are being used (Afla-guard^®^ and AF36^®^) for the prevention of aflatoxin in peanuts, corn, and cotton seed (Dorner, 2009). Good agricultural practices (GAPs) also help control the toxins to a larger extent, such as timely planting, providing adequate plant nutrition, controlling weeds, and crop rotation, which effectively control *A. flavus* infection in the field (Ehrlich and Cotty, 2004; Waliyar et al., 2013).

Biological control is emerging as a promising approach for aflatoxin management in groundnuts using *Trichoderma* spp, and significant reductions of 20–90% infection of aflatoxin have been recorded (Anjaiah et al., 2006; Waliyar et al., 2015). Use of inbred maize lines resistant to aflatoxin has also been employed. Potential biochemical markers and genes for resistance in maize against *Aspergillus* could also be utilized (Chen et al., 2007). Additionally, biotechnological approaches have been reviewed for aflatoxin management strategies (Yu, 2012). Advances in genomic technology based research and decoding of the *A. flavus* genome have supported identification of the genes responsible for production and modification of the aflatoxin biosynthesis process (Bhatnagar et al., 2003; Cleveland, 2006; Holbrook et al., 2006; Ehrlich, 2009). In addition, Wu (2010) suggested that aflatoxin accumulation can be reduced by utilizing transgenic *Bt* maize with insect resistance traits as the wounding caused by insects helps penetrate the *Aspergillus* in kernels.

## Conclusion

Aflatoxins are a major source of disease outbreaks due to a lack of knowledge and consumption of contaminated food and feed worldwide. Excessive levels of aflatoxins in food of non-industrialized countries are of major concern. Several effective physical, chemical, biological, and genetic engineering techniques have been employed for the mitigation, effective control and management of aflatoxins in food. However, developing fungal resistant and insect resistant hybrids/crops to combat pre-harvest infections and their outcome is a major issue of concern. Post-harvest treatments to remove aflatoxins such as alkalization, ammonization, and heat or gamma radiation are not generally used by farmers. However, some of the microorganisms naturally present in soil have the ability to degrade and reduce the aflatoxin contamination in different types of agricultural produce. Therefore, methods of using these organisms to reduce aflatoxin are currently being focused on. Moreover, application of genetic recombination in *A. flavus* and other species is being investigated for its potential to mitigate aflatoxins to ensure the safety and quality of food.

## Author Contributions

PK and DM designed and conceived the experiments and wrote the manuscript. MK, TM, and SK edited and helped in finalizing the manuscript.

## Conflict of Interest Statement

The authors declare that the research was conducted in the absence of any commercial or financial relationships that could be construed as a potential conflict of interest.

## Footnotes

1 https://en.wikipedia.org/wiki/2013_European_aflatoxin_contamination
